# Protective and Exacerbating Cognition and Attribution Factors From the Cognitive Discrepancy Theory of Loneliness

**DOI:** 10.1093/geroni/igab046.150

**Published:** 2021-12-17

**Authors:** Jillian Minahan Zucchetto

**Affiliations:** Fordham University, New York, New York, United States

## Abstract

According to the cognitive discrepancy theory, although the discrepancy between actual and desired social resources may result in loneliness, Perlman and Peplau (1998) suggested that cognitive processing and attributional style also impact the interpretation of social information. Previous empirical research investigating predictors of loneliness have not assessed a wide range of cognition and attribution factors, so this study filled this gap by examining how protective (optimism, sense of mastery, and purpose in life) and exacerbating (depression, control constraints, negative self-perceptions of aging (SPA), and experiences of age-based discrimination) factors influence and moderate the experience of loneliness cross-sectionally and longitudinally using a sample of 3,345 Americans aged 50 years and older from the 2008 and 2012 waves of the Health and Retirement Study. Optimism (βs = -.15, -.13), mastery (βs = -.08, -.07), purpose in life (βs = -.19, -.18), depression (βs = .22,.14), control constraints (βs = .18, .17), negative SPA (βs = .13, .14), and experiences of ageism (βs = .07, .06) were significantly related to loneliness cross-sectionally and longitudinally, respectively. Optimism buffered the negative impact of poor functional social resources (e.g., low social support) on loneliness cross-sectionally while control constraints, negative SPA, and experiencing ageism exacerbated the relationship between low functional social resources and loneliness cross-sectionally. None of the protective or exacerbating factors modulated the relationship between functional social resources and loneliness longitudinally. These findings have important implications for the development of interventions that target loneliness. Targeting maladaptive cognitions may be particularly effective in reducing loneliness.

